# Real-World Effectiveness of Wearable Augmented Reality Device for Patients With Hearing Loss: Prospective Study

**DOI:** 10.2196/33476

**Published:** 2022-03-23

**Authors:** Ul Gyu Han, Jung-Yup Lee, Ga-Young Kim, Mini Jo, Jaeseong Lee, Kyoung Ho Bang, Young Sang Cho, Sung Hwa Hong, Il Joon Moon

**Affiliations:** 1 Samsung Advanced Institute for Health Sciences & Technology, Department of Health Sciences and Technology Sungkyunkwan University Seoul Republic of Korea; 2 Hearing Research Laboratory, Samsung Medical Center Seoul Republic of Korea; 3 Department of Otorhinolaryngology-Head and Neck Surgery, Samsung Medical Center Sungkyunkwan University School of Medicine Seoul Republic of Korea; 4 Advanced Lab - Audio, Samsung Electronics Suwon Republic of Korea; 5 Department of Electrical and Electronic Engineering, Yonsei University Seodaemun-gu, Seoul Republic of Korea; 6 Department of Otorhinolaryngology-Head and Neck Surgery, Samsung Changwon Hospital Sungkyunkwan University School of Medicine Changwon Republic of Korea

**Keywords:** hearing loss, hearing aids, personal sound amplification product, wearable augmented reality device

## Abstract

**Background:**

Hearing loss limits communication and social activity, and hearing aids (HAs) are an efficient rehabilitative option for improving oral communication and speech comprehension, as well as the psychosocial comfort of people with hearing loss. To overcome this problem, over-the-counter amplification devices including personal sound amplification products and wearable augmented reality devices (WARDs) have been introduced.

**Objective:**

This study aimed to evaluate the clinical effectiveness of WARDs for patients with mild to moderate hearing loss.

**Methods:**

A total of 40 patients (18 men and 22 women) with mild to moderate hearing loss were enrolled prospectively in this study. All participants were instructed to wear a WARD, Galaxy Buds Pro (Samsung Electronics), at least 4 hours a day for 2 weeks, for amplifying ambient sounds. Questionnaires including the Korean version of the abbreviated profile of hearing aid benefit (K-APHAB) and the Korean adaptation of the international outcome inventory for hearing aids (K-IOI-HA) were used to assess personal satisfaction in all participants. Audiologic tests, including sound field audiometry, sound field word recognition score (WRS), and the Korean version of hearing in noise test (K-HINT), were administered to 14 of 40 patients. The tests were performed under two conditions: unaided and aided with WARDs.

**Results:**

The mean age of the participants was 55.4 (SD 10.7) years. After 2 weeks of the field trial, participants demonstrated a benefit of WARDs on the K-APHAB. Scores of 3 subscales of ease of communication, reverberation, and background noise were improved significantly (*P*<.001). However, scores regarding aversiveness were worse under the aided condition (*P*<.001). K-IOI-HA findings indicated high user satisfaction after the 2-week field trial. On audiologic evaluation, the K-HINT did not show significant differences between unaided and aided conditions (*P*=.97). However, the hearing threshold on sound field audiometry (*P*=.001) and the WRS (*P*=.002) showed significant improvements under the aided condition.

**Conclusions:**

WARDs can be beneficial for patients with mild to moderate hearing loss as a cost-effective alternative to conventional hearing aids.

## Introduction

### Background

Hearing loss limits communication and social activity, leading to disorders in language and cognitive impairment [1]. According to the World Health Organization, approximately 5% of the world’s population has hearing loss, and the number is anticipated to increase to one in every four people by 2050 because of rapidly aging populations [2]. Oral communication is crucial for contact with other people, but people with hearing loss have reduced speech understanding compared to people with normal hearing. Therefore, active hearing rehabilitation is needed for people with hearing loss [3].

Hearing aids (HAs) are an efficient rehabilitative option for improving oral communication and speech comprehension as well as the psychosocial comfort of people with hearing loss [4]. Although the benefits of HAs have been well documented, the uptake rate of HAs remains relatively low [5]. Furthermore, only 0.47% of individuals with minimal hearing loss use HAs even when experiencing subjective hearing difficulty [6]. One of the main reasons for low uptake of HAs is high cost. The average set of HAs costs from US $1000-$5000, which can inflict a financial burden on many individuals with hearing loss [7]. According to the MarkeTrack VIII survey, some consumers with mild to moderate hearing loss said that they would adopt HAs if the price did not exceed a certain level or if they were covered by insurance [8].

To overcome this problem, over-the-counter (OTC) amplification devices including personal sound amplification products (PSAPs) have been introduced. PSAPs are defined by the US Food and Drug Administration as wearable electronic products for customers with hearing loss to amplify sounds in certain environments. In general, PSAPs are less expensive and simpler sound amplification devices with fewer features and less functionality than digital HAs. However, some studies have suggested some kinds of PSAPs as alternative devices for those with mild to moderate hearing loss [9]. In addition, we reported that wearable augmented reality devices (WARDs) with a broad spectrum of “hearable” have the potential to be beneficial for individuals with hearing loss. WARDs are a combination of smartphone apps and earbuds, providing a personalized listening experience. For example, the Samsung Galaxy Buds Pro has its own smartphone app called Galaxy Wearable. Users can take advantage of a feature called ambient sound. Similar to PSAPs, individuals can manage the level of sounds in their surroundings such as crowded restaurant or sidewalk with many cars. They can also reduce background noise and listen to music on the street or subway. WARDs helped people with mild to moderate hearing loss to understand conversations in quiet environments [10].

### Objectives

Although most previous studies evaluated clinical effectiveness of PSAPs compared to conventional HAs, there are insufficient data on the WARD’s ability to help people with hearing loss. To the best of our knowledge, no clinical field trial assessing the effectiveness of WARDs in the daily lives of hearing-impaired people has been conducted. Thus, the aims of this study were to investigate the hearing outcomes in patients with mild to moderate hearing loss aided with WARDs and to quantify the patient’s subjective outcomes using the Korean version of the abbreviated profile of hearing aid benefit (K-APHAB) and the Korean adaptation of the international outcome inventory for hearing aids (K-IOI-HA) questionnaires in 2-week field trials. Furthermore, we attempted to assess the correlation between personal satisfaction and audiologic performance with WARDs.

## Methods

### Participants

The sample size was determined on the basis of previous research determining the effect of a web-based intervention program on positive changes in hearing aid use [11]. The resulting sample size was 21, using G*Power 3.1.9.7 for power set at 0.95 and α set at .05. A total of 40 individuals with mild to moderate hearing loss were enrolled in the study. A prospective study was conducted with subjects who visited the outpatient clinic of the department of otolaryngology for hearing loss from February to May 2021. The subjects who met the following appropriateness criteria were included: patients between 18 and 70 years of age who had bilateral mild to moderate hearing loss (26-55 dB hearing level [HL]; pure tone average 500-4000 Hz) and who were determined to have no abnormalities in the eardrum on otoscopy. The exclusion criteria were difficulty of communication or inspection and inability to handle the device.

### Ethical Considerations

This study was approved by the institutional review board of Samsung Medical Center in Seoul, South Korea (2020-05-052, 2020-10-163), and conducted in accordance with the Declaration of Helsinki. Informed consent was obtained from all participants.

### Intervention

Galaxy Buds Pro (SM-R175, Samsung Electronics) was used for hearing rehabilitation in this study. Galaxy Buds Pro has its own smartphone app, Galaxy Wearable. Users can use the Ambient Sound feature with the app for sound amplification. Similar to PSAPs or HAs, users can control the level of sound in their surroundings using Galaxy Buds Pro. The Galaxy wearable device consists of 4 levels, of which only level 4 provided sound amplification. Therefore, the level was set at 4 in this study.

Each participant was provided with a pair of Galaxy Buds Pro for this field trial and taught how to use the device. Participants were required to use the device when having difficulties in communication or listening and for more than 4 hours a day for 2 weeks. They wore the device during daily activity such as conversation, TV watching, or driving.

We also recommended to stop wearing the devices when if the participants feel any pain or have any other troubles in their ears. Before the beginning of the field trial, participants filled out the K-APHAB questionnaire as a baseline [12].

**Figure 1:**
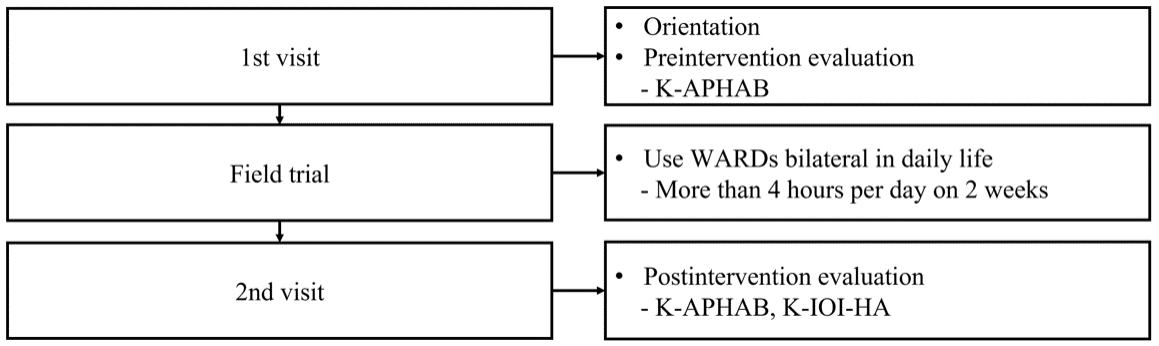
Flowchart of the 2-week field study. Subjects were required to use the WARD more than 4 hours a day for 2 weeks. At the second visit, questionnaires were completed by all subjects. K-APHAB: Korean version of abbreviated profile of hearing aid benefit, K-IOI-HA: Korean adaptation of the international outcome inventory for hearing aids, WARD: wearable augmented reality device.

After 2 weeks, participants returned the device and filled out the K-APHAB and the K-IOI-HA questionnaires to assess the benefit of using the device [13]. The APHAB is one of the most commonly used questionnaires to assess the benefit of HAs and often is cited for its ease of understanding and delivery [14]. The K-APHAB consists of 24 questions divided into 4 subscales that measure hearing loss in everyday situations. The ease of communication (EC) subscale examines basic hearing situations without ambient noise in a quiet environment, the background noise (BN) subscale examines hearing situations with background noise, the reverberation (RV) subscale investigates hearing situations in large spaces with echoes, and the aversiveness (AV) subscale measures the perception of loud sound events [15]. Global scores are calculated as the average of the EC, BN, and RV subscale scores [16]. Higher scores reflect a greater rate of problems. In general, HA benefit as indicated by K-APHAB is calculated as unaided scores minus aided scores and is represented by a positive value. We utilized the K-APHAB for evaluating Galaxy Buds Pro benefits. The IOI-HA was designed to formulate a standardized and internationally useful self-report measurement. A self-report measurement is necessary to acquire quantifiable data on the effects of HAs in users’ daily lives [17]. Similar to the APHAB, the IOI-HA has been utilized to investigate an aspect of the personal impact of hearing rehabilitation devices [18]. The K-IOI-HA contains seven questions used to subjectively evaluate HA performance using these parameters: (1) duration of HA use (USE), (2) benefit (BEN), (3) residual limitation in daily life activities (RAL), (4) satisfaction (SAT), (5) residual restrictions to participation (RPR), (6) impact on other people (IO), and (7) quality of life (QOL). Patients select one of five responses. Therefore, each question can be scored from 1 to 5 points, and the total score ranges from 7 to 35 points, with a high score indicating a positive HA effect. Furthermore, we divided two subscales (Factors 1 and 2) within the K-IOI-HA when performing a principal component analysis. Factor 1 included USE, BEN, SAT, and QOL; Factor 2 included RAL, RPR, and IO. Factor 1 described the overall benefit with WARD, and Factor 2 described the residual limitations after WARD fitting [19]. We utilized the K-IOI-HA for evaluating an outcome with Galaxy Buds Pro. A flowchart of the 2-week field study is presented in Figure 1.

### Audiologic Evaluation

Three audiologic test batteries were administered in this study: (1) sound field audiometry, (2) sound-field word recognition score (WRS), and (3) the Korean version of hearing in noise test (K-HINT). The associated measurements were conducted with 14 subjects who agreed to participate in accordance with institutional review board approval.

Unaided and aided thresholds were obtained in sound field audiometry. Warble tones of 0.25, 0.5, 1, 2, 3, 4, and 6 kHz were presented through a loudspeaker located 1 m from the participant. The participant wore the WARD in both ears to mimic how the device would be used in his/her daily life. Speech perception with and without the WARD was evaluated through sound-field WRS. In total, 25 monosyllabic words from the Korean standard monosyllabic word list (KS-MWL-A) were presented at 50 dB HL through a loudspeaker located 1 m from the participant [20]. The participant was asked to repeat the word back to the tester. The percentage of correct scores was calculated. Last, K-HINT was performed to assess speech recognition in the presence of noise. The participant sat on a chair in the center of the sound field, facing a loudspeaker that was located approximately 1 m away at the 0˚ azimuth. The target sentences in the K-HINT and speech-shaped noise were presented by the loudspeaker at a fixed level of 65 dBA. The presentation level of the target speech was adjusted to measure a signal-to-noise ratio (SNR) at which the participant recognized the sentences 50% of the time.

### Statistical Analysis

All statistical analyses were completed using SPSS (version 26; IBM Corp). The paired 2-tailed *t* test was conducted to compare the scores of questionnaires before and after intervention. The paired *t* test also was used to compare the variables of audiological measurements between unaided and aided conditions. In addition, Pearson correlation coefficients were calculated to further investigate relationships between scores on the questionnaires and laboratory assessments. A significance level of *P*=.05 was applied to determine statistical significance.

## Results

### Demographics

**Figure 2:**
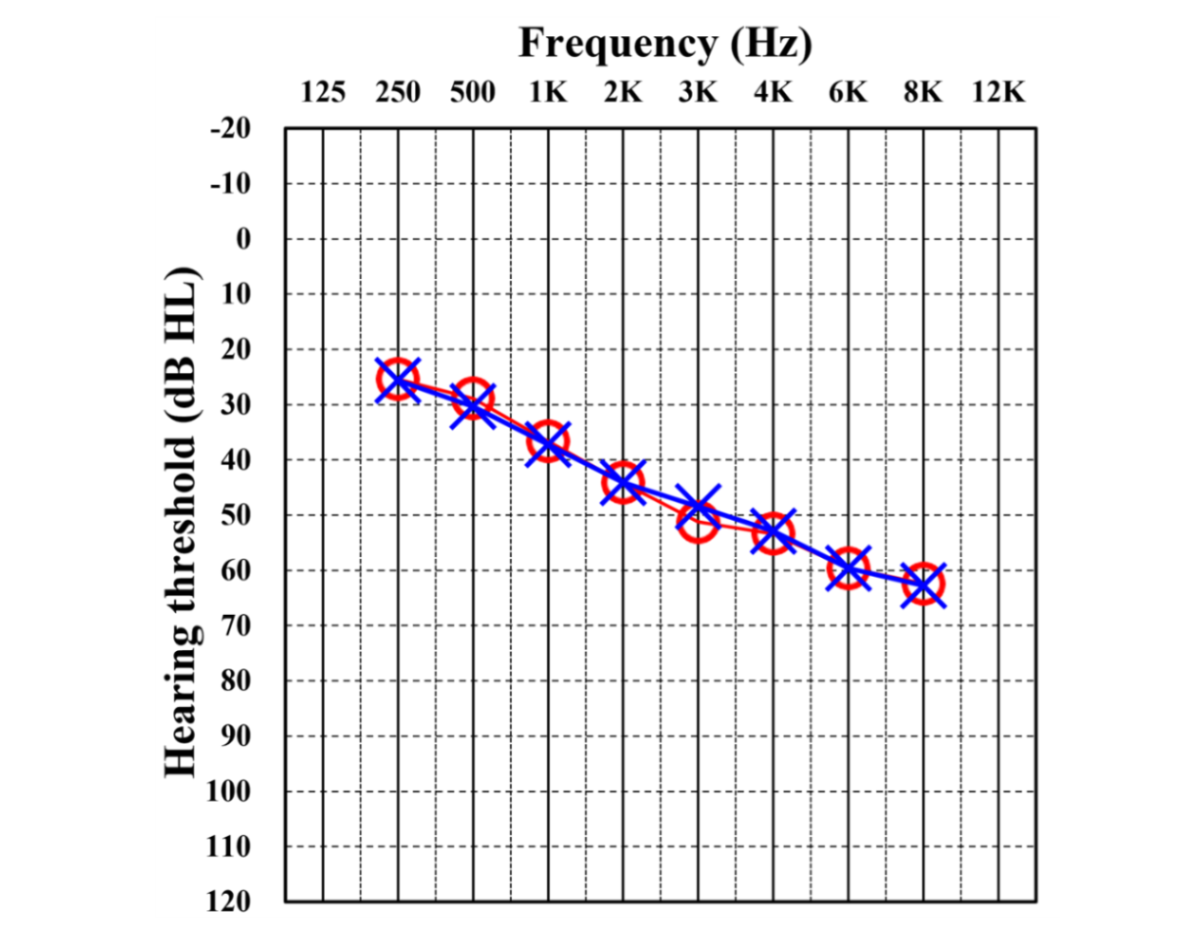
Audiogram representing the mean hearing threshold of all participants (N=40). Hearing threshold is the minimum volume of sound that participant can hear at a specific frequency, indicated by red “O” (right ear) or blue “X” (left ear). HL: hearing level.

A total of 40 participants (18 male and 22 female; mean age 55.4, SD 10.73, range 28-67 years) with mild to moderate hearing loss were enrolled. The mean hearing threshold in pure-tone average was 40.75 (SD 6.63) dB on the right side and 41.16 (SD 7.93) dB on the left side (Figure 2).

The demographic characteristics of the enrolled patients are summarized in Table 1. If previous usage durations of HAs or PSAPs in each ear were different, the average usage duration of the 2 ears was calculated.

**Table 1:** Demographic information of all participants (N=40).

Variable		Value
Age (years), mean (SD)		55.4 (10.73)
**Sex, n (%)**		
	Male	18 (45)
	Female	22 (55)
**Hearing threshold (dB hearing level), mean (SD)**		
	Right	40.75 (6.63)
	Left	41.16 (7.93)
**Previous hearing aid users**		
	Direction (Unilateral:Bilateral), n	3:4
	Type (invisible in canal:receiver in canal:in the canal:receiver in ear:complete in canal), n	2:2:1:1:1
	Usage duration (months), mean (SD)	8.57 (9.07)
**Previous personal sound amplification product user, n**		
	Direction (Unilateral:Bilateral), n	2:0
	Type (complete in canal:receiver in canal), n	1:1
	Usage duration (months), mean (SD)	2.25 (1.77)

### Questionnaires

**Figure 3:**
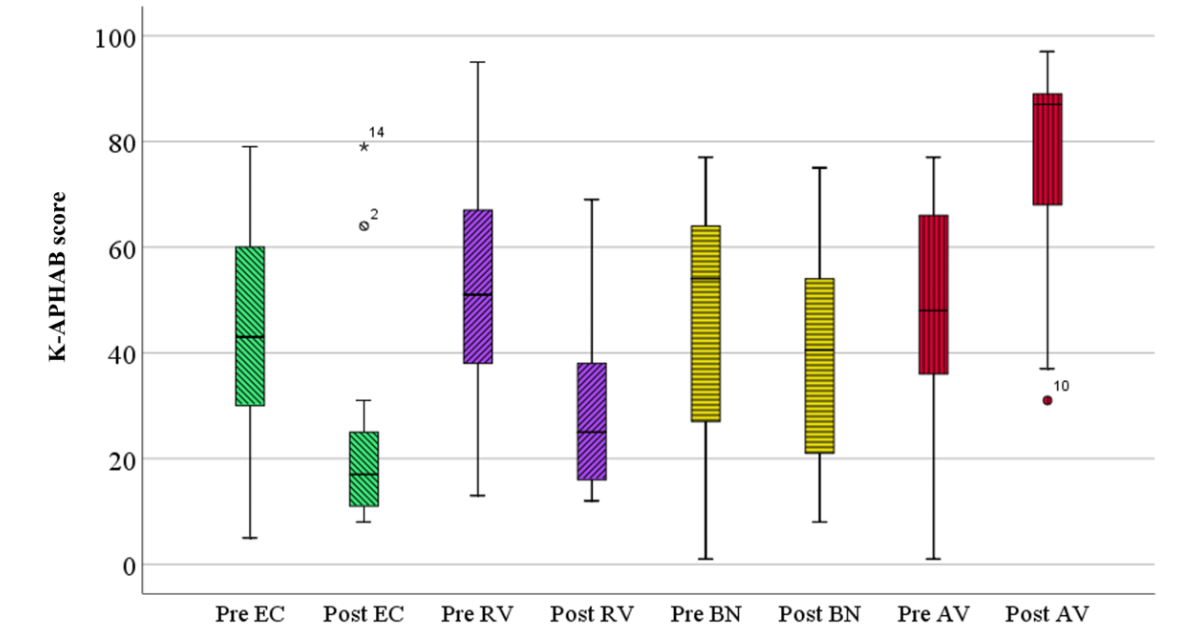
The results of the K-APHAB. There were significant reductions in the subscales of EC, RV, and BN (*P*<.001). In contrast, significant increase in the subscale of AV was observed in the aided condition as compared to the unaided condition (*P*<.001). AV: aversiveness, BN: background noise, EC: ease of communication, K-APHAB: Korean version of abbreviated profile of hearing aid benefit, Post: aided, Pre: unaided, RV: reverberation.

The K-APHAB results for all 40 subjects under unaided and aided conditions are shown in Figure 3. The EC subscale under unaided (Pre EC) and aided conditions (Post EC) was 42.30 points (SD 21.04) and 20.15 points (SD 14.56), respectively. The EC subscale was significantly decreased with the WARD (*P*<.001). The RV subscale before using the WARD (Pre RV) was 51.58 points (SD 20.49). Under the aided condition, the RV subscale (Post RV) was 27.35 (SD 13.97) points, which was significantly improved (*P*<.001). There was a significant difference between the BN subscale under unaided (Pre BN) and aided conditions (Post BN) (*P*<.001). Pre BN score was 48.28 (SD 18.75) points, while the Post BN score was 35.17 (SD 18.18) points. Additionally, the global score (average of EC, RV, and BN subscales) showed significant improvement under the aided condition compared with the unaided condition (*P*<.001). In contrast, the AV subscale score in the aided condition (Post AV) was worse than that in the unaided condition (Pre AV). There was a significant difference between Pre AV points and Post AV points (*P*<.001).

**Figure 4:**
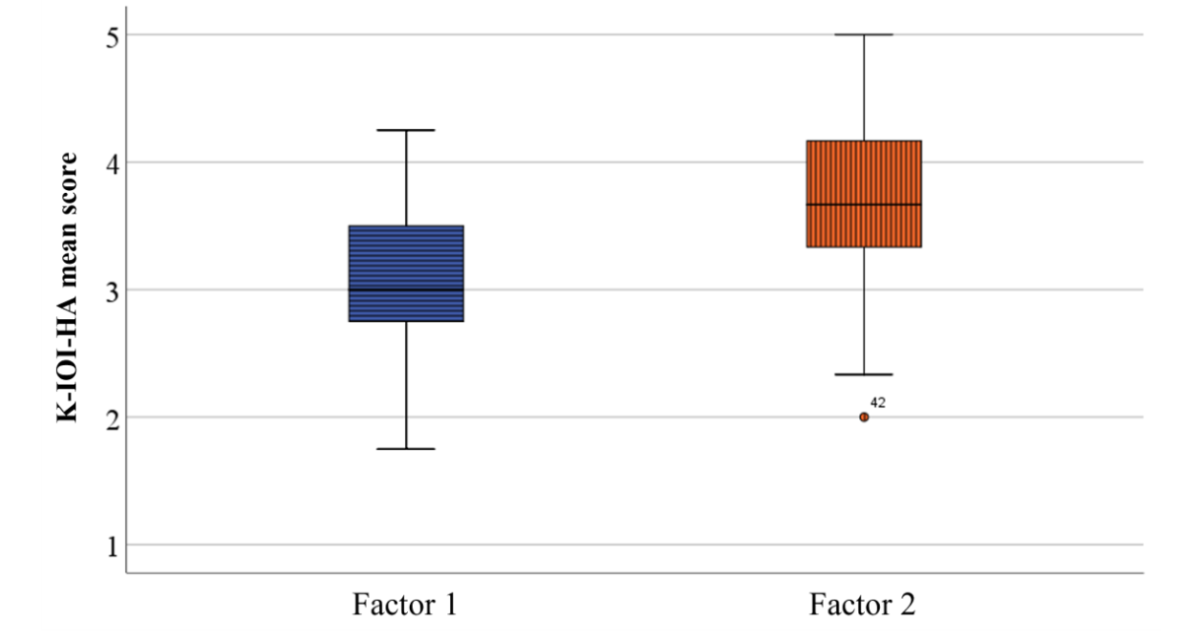
The comparison of the mean score on K-IOI-HA Factor 1 and Factor 2. There was a significant difference between the mean score of Factor 1 (total score of USE, BEN, SAT, and QOL) and the mean score of Factor 2 (total score of RAL, RPR, and IO) (*P*<.001). BEN: benefit, Factor 1: sum score of the USE (daily use), Factor 2: sum score of the RAL (residual activity limitations), IO: impact on others, K-IOI-HA: Korean adaptation of the international outcome inventory for hearing aids; QOL: quality of life, RPR: residual participation restriction, SAT: satisfaction (with the device and services).

The resulting scores on the K-IOI-HA for the 40 subjects were 3.3 (SD 0.5) points for daily USE, 3.0 (SD 0.9) points for BEN, 3.6 (SD 0.9) points for RAL, 3.0 (SD 1.0) points for SAT with devices and services, 3.7 (SD 0.9) points for RPR, 4.2 (SD 0.9) points for IO, and 3.0 (SD 0.7) points for QOL. In addition, the mean K-IOI-HA Factor 2 score was significantly higher than the Factor 1 score (*P*<.001; Figure 4).

### Audiologic Measurements

For 14 participants, the average threshold of sound field audiometry under the unaided condition was 43.45 (SD 6.62) dB HL; this was significantly decreased under the aided condition to 40.48 (SD 6.99) dB HL (*P*=.001). Sound field WRS significantly improved in the aided condition from 55.43% (SD 21.45%; responded to ~14 of 25 test questions) to 67.71% (SD 16.11%; responded to ~17 of 25 test questions) (*P*=.002). However, there was no significant differences in the K-HINT score between the unaided and aided conditions (Table 2).

**Table 2:** Results of audiologic measurements (N=14).

Audiologic measurements	Unaided condition	Using the Galaxy Buds Pro wearable augmented reality device	*P* value
Sound field audiometry threshold^a^ (dB hearing level), mean (SD)	43.45 (6.62)	40.48 (6.99)	.001
Word recognition score (%), mean (SD)	55.43 (21.45)	67.71 (16.11)	.002
Korean version of hearing in noise test (dB signal-to-noise ratio), mean (SD)	–0.65 (1.77)	–0.68 (2.10)	.97

^a^Average of hearing thresholds at six frequencies: 0.5, 1, 1, 2, 2, and 4 kHz.

### Correlations Between Audiologic Measurements and Patient-Reported Outcome Measurements

**Figure 5:**
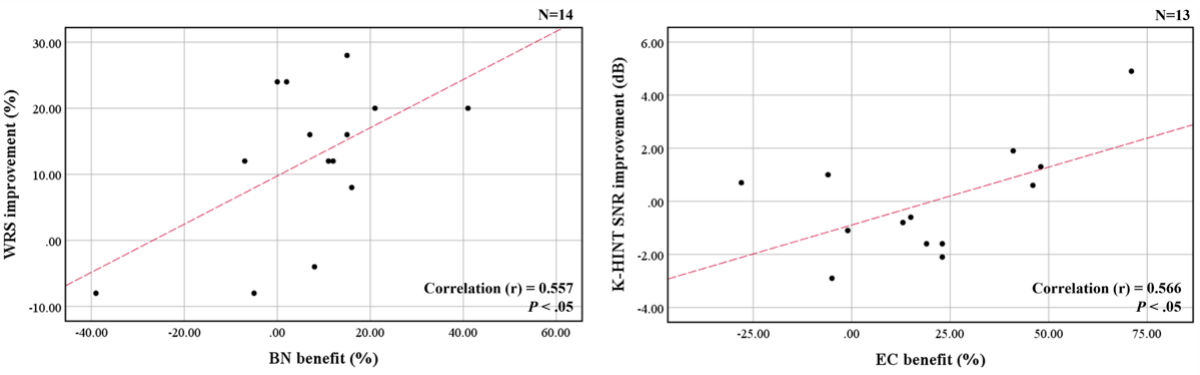
Correlation between audiologic parameters and personal satisfaction. BN and EC benefit on the K-APHAB each a showed significant correlation with WRS improvement and SNR improvement on the K-HINT. WRS improvement: aided WRS – unaided WRS. BN benefit: aided BN – unaided BN. EC benefit: aided EC – unaided EC. BN: background noise, EC: ease of communication, K-APHAB: Korean version of abbreviated profile of hearing aid benefit, K-HINT: Korean version of hearing in noise test, SNR: signal-to-noise ratio, WRS: word recognition score.

Correlation analysis between scores from questionnaire and audiologic evaluation was performed (Figure 5). The EC benefit on the K-APHAB questionnaire showed a significant correlation with dB SNR improvement on the K-HINT (*P*<.05). BN benefit also showed a significant correlation with WRS improvement (*P*<.05). However, EC benefit did not show a significant correlation with WRS improvement (*P*=.99), and BN benefit did not show a significant correlation with dB SNR improvement on the K-HINT (*P*=.20). No additional correlations were found between the parameters.

## Discussion

### Principal Findings

This study investigated personal satisfaction with the WARD among patients with mild to moderate hearing loss. Furthermore, the performance of the WARD was evaluated through audiologic tests including sound field audiometry, WRS, and the K-HINT. The results revealed significantly increased subjective satisfaction with the WARD. Furthermore, sound field audiometry and WRS also showed significant improvement under the aided condition.

In this study, significant improvements were shown in EC, RV, and BN subscales under aid with a WARD. These results indicated that if patients use a WARD, their difficulties in listening were improved in various situations including quiet or easy conversation, loud sounds or sound at a distance, and in the presence of ambient noise. However, the AV subscale was significantly increased under the aided condition: loud and noisy sounds were amplified with a WARD with a resulting increase in discomfort.

Worse scores on the AV subscale occur commonly, even in HA users. Johnson et al [21] reported that AV benefit decreased under both conditions, aided with linear processing type HAs and wide dynamic processing compression HAs. In another study, the AV subscale score was increased under the aided condition with all types of HAs, including those completely in the canal, in the canal, and behind the ear [22]. Ideally, patients with hearing impairment would show increased EC, RV, BN, and AV benefit when wearing HAs. However, various studies have shown that AV benefit is difficult to achieve even with high-quality HAs [23]. In addition to amplifying environmental noise, lack of acclimatization or individual fine fitting can be reasons for this unsatisfactory result [24]. WARDs cannot be tuned individually, which is a limitation of the device. Therefore, further technical development such as individual fitting or advanced artificial intelligence system would be needed to overcome those problems.

In this study, the average of all question scores was 3.0 points or higher. In particular, the IO score was higher than 4 points, indicating decreased inconvenience to others owing to hearing difficulty when using the WARD. Compared with previously published IOI-HA norms, the distribution characteristics of the mean scores were consistent with the normative data [25]. The comparison also indicated that our scores were slightly higher than the normative data for IO score.

The average threshold of sound field audiometry was significantly improved with WARDs in this study. Sound field WRS was increased while wearing a WARD. Thus, hearing-impaired people can receive listening benefit from WARDs under quiet conditions. However, speech recognition in a noisy environment was worse under the aided condition. Reed at al [26] reported that PSAPs improved speech recognition in a noisy environment for those with mild to moderate hearing loss. However, the results of this study were insufficient to demonstrate the benefit of a WARD for improvement of speech intelligibility in a noisy environment. Clarifying the reason for the difference of these results is difficult, and the effectiveness of WARDs in the noisy environment remains uncertain.

Interpreting the correlation between objective and subjective results requires caution. We analyzed the correlation between benefits from K-APHAB scores and parameters from audiologic measurements in this study. These were not significantly correlated, but there were some weak correlations between personal satisfaction and audiologic performance. We confirmed that the improvement in dB SNR on the K-APHAB and the EC benefit on the K-APHAB and that between the BN benefit and the WRS on the K-APHAB had significant correlations (*P*<.05). We expected the WRS improvement and EC benefit to have a significant correlation because both were derived in a quiet environment. We also expected that dB SNR improvement and BN benefit would have a significant correlation because both results were derived in a noisy environment. However, some results in this study did not agree with our expectations. The group with high satisfaction in a quiet environment showed dB SNR improvement, and the group with high satisfaction in a noisy environment had WRS improvement.

The reason for this correlation analysis can be considered to be due to the difference in the test conditions. First, as mentioned in previous studies, the laboratory-based evaluation method is a static, limited, and 1-way communication method, whereas the evaluation method experienced by patients in everyday life is a dynamic and expanded interactive communication method [27]. Second, the difference between sound and noise should be considered. In the test room, subjects heard the voice of one speaker at a certain volume of speech spectrum noise. However, in everyday life, subjects hear the voices of one or more speakers with a variety of noises. Third, in everyday life, various visual stimuli can be referenced for sound recognition; this was not the case under our test conditions. As such, difficulty arises in considering various factors that can affect the evaluation results in a laboratory environment. Therefore, a number of studies is being conducted to consider various conditions in daily life via VR technology [27,28].

Overall satisfaction with a WARD in daily life was high in this study, and we speculate that this high satisfaction might be influenced by the current COVID-19 pandemic. All Koreans must wear face masks when outdoors. Personal protective equipment such as the facial mask creates difficulty in understanding and communication in hearing impaired patients; these patients cannot read the speaker’s lips, and sound clarity is decreased [29]. In this pandemic situation, WARDs could foster conversations.

### Limitations

This study has some limitations. The first limitation is the lack of generalizability to the WARDs market. Only one kind of WARD, Galaxy Buds Pro, was used in this study. Even though availability of hearing devices is limited, WARDs vary considerably in style, quality, and technology. Therefore, further research using a variety of hearable devices is needed to generalize the feasibility of WARDs. Second, we did not take into consideration certain subject demographics including the duration of hearing loss, education, income, and perceived social support, which play an important role in determining personal satisfaction. Future large-scale research should consider the contribution of these factors to audiologic outcomes and personal satisfaction related to WARD benefit. Finally, we did not take into account the shape and size of the ear canal. Since the shape and size of human ear canal are very diverse, it might be possible that there were some participants who had WARDs that were either too large or too small [30]. Even though there was no participant who complained about it, the shape and size of WARDs would also be a problem worth investigating.

### Conclusions

Our results indicate that WARDs could be helpful for individuals with mild to moderate hearing loss, especially under quiet conditions. Owing to high price and poor accessibility of HAs, OTC hearing devices such as WARDs could be an alternative partial solution for hearing loss. In the near future, WARDs will have greater potential as technology develops and government regulation changes. Further large-scale comparative research regarding the clinical effectiveness of WARDs is necessary.

## Abbreviations

AV: aversiveness
BEN: benefit
BN: background noise
EC: ease of communication
HA: hearing aid
HL: hearing level
IO: impact on other people
K-APHAB: Korean version of the abbreviated profile of hearing aid benefit
K-HINT: Korean version of hearing in noise test
K-IOI-HA: Korean adaptation of the international outcome inventory for hearing aids
OTC: over the counter
Post AV: aversiveness subscale under the aided condition
Post BN: background noise subscale under the aided condition
Post EC: ease of communication subscale under aided condition
Post RV: reverberation subscale under the aided condition
Pre AV: aversiveness subscale under the unaided condition
Pre BN: background noise subscale under the unaided condition
Pre EC: ease of communication subscale under the unaided condition
Pre RV: reverberation subscale before using the wearable augmented reality device
PSAP: personal sound amplification product
QOL: quality of life
RAL: residual limitation in daily life activities
RPR: residual restrictions to participation
RV: reverberation
SAT: satisfaction
USE: duration of HA use
WARD: wearable augmented reality device
WRS: word recognition score

## References

[1] Dalton DS, Cruickshanks KJ, Klein BEK, Klein R, Wiley TL, Nondahl DM (2003). The impact of hearing loss on quality of life in older adults. Gerontologist.

[2] (2021). WHO: 1 in 4 people projected to have hearing problems by 2050. World Health Organization.

[3] Li LYJ, Wang S, Wu C, Tsai C, Wu T, Lin Y (2020). Screening for hearing impairment in older adults by smartphone-based audiometry, self-perception, HHIE screening questionnaire, and free-field voice test: comparative evaluation of the screening accuracy with standard pure-tone audiometry. JMIR Mhealth Uhealth.

[4] Hughes ME, Nkyekyer J, Innes-Brown H, Rossell SL, Sly D, Bhar S, Pipingas A, Hennessy A, Meyer D (2018). Hearing aid use in older adults with postlingual sensorineural hearing loss: protocol for a prospective cohort study. JMIR Res Protoc.

[5] Nkyekyer J, Meyer D, Blamey PJ, Pipingas A, Bhar S (2018). Investigating the impact of hearing aid use and auditory training on cognition, depressive symptoms, and social interaction in adults with hearing loss: protocol for a crossover trial. JMIR Res Protoc.

[6] Choi JE, Ahn J, Park HW, Baek S, Kim S, Moon IJ (2017). Prevalence of minimal hearing loss in South Korea. PLoS One.

[7] What to Know About Current Hearing Aid Costs and Pricing. Healthline.

[8] Kochkin S (2000). MarkeTrak V: Consumer satisfaction revisited. Hear J.

[9] Cho YS, Park SY, Seol HY, Lim JH, Cho Y, Hong SH, Moon IJ (2019). Clinical performance evaluation of a personal sound amplification product vs a basic hearing aid and a premium hearing aid. JAMA Otolaryngol Head Neck Surg.

[10] Seol HY, Kim G, Kang S, Jo M, Han UG, Cho YS, Hong SH, Moon IJ (2021). Clinical comparison of a hearing aid, a personal sound amplification product, and a wearable augmented reality device. Clin Exp Otorhinolaryngol.

[11] Thorén ES, Oberg M, Wänström G, Andersson G, Lunner T (2014). A randomized controlled trial evaluating the effects of online rehabilitative intervention for adult hearing-aid users. Int J Audiol.

[12] Lim HJ, Park MK, Cho Y, Han GC, Choi J, An Y, Kim BJ, Choi BY (2017). Validation of the Korean version of the abbreviated profile of hearing aid benefit. Korean J Otorhinolaryngol-Head Neck Surg.

[13] Chu H, Cho Y, Park S, Byun JY, Shin JE, Han GC, Cheon BC, Lee JH, Jung JY (2012). Standardization for a Korean adaptation of the International Outcome Inventory for Hearing Aids: study of validity and reliability. Korean J Otorhinolaryngol-Head Neck Surg.

[14] Cox R, Hyde M, Gatehouse S, Noble W, Dillon H, Bentler R, Stephens D, Arlinger S, Beck L, Wilkerson D, Kramer S, Kricos P, Gagné JP, Bess F, Hallberg L (2000). Optimal outcome measures, research priorities, and international cooperation. Ear Hear.

[15] Cox RM, Alexander GC (1995). The abbreviated profile of hearing aid benefit. Ear Hear.

[16] Dornhoffer J, Meyer T, Dubno J, McRackan T (2020). Assessment of hearing aid benefit using patient-reported outcomes and audiologic measures. Audiol Neurootol.

[17] Cox RM, Alexander GC (2002). The International Outcome Inventory for Hearing Aids (IOI-HA): psychometric properties of the English version. Int J Audiol.

[18] Atas A, Tutar H, Gunduz B, Bayazıt YA (2014). Vibrant SoundBridge application to middle ear windows versus conventional hearing aids: a comparative study based on international outcome inventory for hearing aids. Eur Arch Otorhinolaryngol.

[19] Cox RM, Stephens D, Kramer SE (2002). Translations of the International Outcome inventory for Hearing Aids (IOI-HA). Int J Audiol.

[20] Kim J, Lee J, Lee KW, Bahng J, Lee JH, Choi C, Cho SJ, Shin EY, Park J (2015). Test-retest reliability of word recognition score using Korean standard monosyllabic word lists for adults as a function of the number of test words. J Audiol Otol.

[21] Johnson JA, Cox RM, Alexander GC (2010). Development of APHAB norms for WDRC hearing aids and comparisons with original norms. Ear Hear.

[22] Quintino CA, Mondelli MFCG, Ferrari DV (2010). Directivity and noise reduction in hearing aids: speech perception and benefit. Braz J Otorhinolaryngol.

[23] Skarzynski H, Lorens A, Piotrowska A, Anderson I (2006). Partial deafness cochlear implantation provides benefit to a new population of individuals with hearing loss. Acta Otolaryngol.

[24] Penteado SP, Bento RF, Battistella LR, Silva SM, Sooful P (2014). Use of the satisfaction with amplification in daily life questionnaire to assess patient satisfaction following remote hearing aid adjustments (telefitting). JMIR Med Inform.

[25] Cox RM, Alexander GC, Beyer CM (2003). Norms for the international outcome inventory for hearing aids. J Am Acad Audiol.

[26] Reed NS, Betz J, Kendig N, Korczak M, Lin FR (2017). Personal sound amplification products vs a conventional hearing aid for speech snderstanding in noise. JAMA.

[27] Hohmann V, Paluch R, Krueger M, Meis M, Grimm G (2020). The virtual reality lab: realization and application of virtual sound environments. Ear Hear.

[28] Seol HY, Kang S, Lim J, Hong SH, Moon IJ (2021). Feasibility of virtual reality audiological testing: prospective study. JMIR Serious Games.

[29] Trecca EM, Gelardi M, Cassano M (2020). COVID-19 and hearing difficulties. Am J Otolaryngol.

[30] Kates JM (1988). A computer simulation of hearing aid response and the effects of ear canal size. J Acoust Soc Am.

